# Supplementary material for: Genomic architecture of haddock (Melanogrammus aeglefinus) shows expansions of innate immune genes and short tandem repeats
Source: BMC Genomics. 2018 Apr 10;19:240. doi: 10.1186/s12864-018-4616-y (PMC5894186; doi:10.1186/s12864-018-4616-y)
Supplement: Supplementary file 1 — Note S1 and Table S1-S10. (PDF 595 kb) [file 12864_2018_4616_MOESM1_ESM.pdf]

Supplementary to Genomic architecture of codfishes featured by expansions of innate immune genes and short tandem repeats

Ole K. Tørresen, Marine S. O. Brieuc, Monica H. Solbakken, Elin Sørhus, Alexander J. Nederbragt, Kjetill S. Jakobsen, Sonnich Meier, Rolf B. Edvardsen, Sissel Jentoft

Note S1: The quality of melAeg compared to GM_CA454PB

The melAeg assembly was created in a process very similar to GM_CA454PB, in that raw, i.e. uncorrected, PacBio reads were assembled together with sequences of lesser error rate, Illumina and Illumina/454, respectively. For GM_CA454PB, 454 type mate pairs were included in the assembly process itself, but since Celera Assembler does not handle Illumina type mate pair (which is contaminated with paired end reads of opposite orientation and shorter insert size), these were not included in the melAeg assembly. For both assemblies, strict criteria were set for the unitigs processed in the assemblies. During assembly, unitigs are marked as unique or repeats, and repeat unitigs are only used for scaffolding if they can be placed by paired reads properly.

To be marked as unique, these rules had to be fulfilled. The first rule that triggers marks the unitig:

For GM_CA454PB a single PacBio read could not span more than 90 % of the unitig, the read coverage could be less than 3 in only up to 15 % of the length of the unitig, it had to contain at least 200 reads or had to be at least 10 kbp long.

For melAeg a single PacBio read could not span more than 80 % of the unitig, it had to be in the 80 % of unitigs with the most reads or the unitig had to be 15 kbp long.

These rules were set to get rid of spurious unitigs. Unitigs with one PacBio read fully covering it might pick up many reads that had only a couple of 454/Illumina reads, and therefore the

span restriction was set. If more than 15 % of the unitig was covered with less than 3 reads, this would be quite poor sequence, and of probably little usage. At least 200 bp reads would get rid of many of the same unitigs, those with low coverage or short. The 80 % of unitigs with the most reads function in much the same way, if a unitigs with less than X amount of reads are in the set of unitigs containing 20 % of the reads, it would not be marked as unique. If none of those rules trigger, the unitig had to be either 10 kbp or 15 kbp long to be marked as unique.

There are about 1,500 contigs shorter than 10 kbp in both assemblies, so the lengths are not a strict cut-off. However, this strict cut-off might have excluded some unitigs with useful sequence, such as the large multiple multi-copy gene families investigated there. The longer PacBio reads used for the melAeg assembly should have helped alleviate this issue. In conclusion, the proportion of *MHCI* genes found in melAeg and gadMor2 is as expected from previous studies (Malmstrøm et al., 2016) that is, about 50 – 75 % of expected, depending on how it is counted.

Table S1. The content of Orthogroups_SpeciesOverlaps.csv from OrthoFinder, shows the number of orthogroups shared by each species-pair (each species contain at least one member of each group).

	Cave fish	Zebrafish	(gadMor2)	(gadMor1)	Stickleback	Spotted gar	haddock	Tilapia	Medaka	Amazon molly	Fugu	Tetraodon	Platyfish
Cave fish	**14275**	13356	12019	12433	12498	12752	11037	12551	12014	13220	11974	11990	12968
Zebrafish	13356	**14351**	12162	12577	12650	12906	11160	12693	12155	13417	12077	12050	13110
Atlantic cod (gadMor2)	12019	12162	**13742**	12092	11874	11681	11500	11769	11418	12431	11293	11309	12132
Atlantic cod (gadMor1)	12433	12577	12092	**13753**	13007	12091	11052	12296	11883	12937	11901	11852	12767
Stickleback	12498	12650	11874	13007	**13826**	12139	10855	12466	12095	13141	12023	11978	12987
Spotted gar	12752	12906	11681	12091	12139	**13700**	10762	12221	11683	12884	11652	11628	12591
Atlantic haddock	11037	11160	11500	11052	10855	10762	**12793**	10768	10440	11372	10312	10370	11115
Tilapia	12551	12693	11769	12296	12466	12221	10768	**13494**	12026	13155	12065	11972	12961
Medaka	12014	12155	11418	11883	12095	11683	10440	12026	**13049**	12630	11597	11613	12395
Amazon molly	13220	13417	12431	12937	13141	12884	11372	13155	12630	**14689**	12384	12418	13898
Fugu	11974	12077	11293	11901	12023	11652	10312	12065	11597	12384	**12726**	11849	12279
Tetraodon	11990	12050	11309	11852	11978	11628	10370	11972	11613	12418	11849	**12905**	12284

Platyfish	12968	13110	12132	12767	12987	12591	11115	12961	12395	13898	12279	12284	**14212**

Table S2: Number of STRs in the different assemblies

Species	Assembly size (Mbp)	Number of STRs	loci/Mbp
Amazon molly	749	333,865	445.7
Atlantic cod	644	760,215	1,180.5
Haddock	653	763,036	1,168.5
Cavefish	1,191	672,579	564.7
Fugu	393	181,766	462.5
Medaka	869	190,422	219.1
Platyfish	730	185,784	254.5
Spotted gar	946	114,926	121.5
Stickleback	462	236,110	511.1
Tetraodon	359	227,056	632.5
Tilapia	927	300,727	324.4
Zebrafish	1,372	1,037,400	756.1

Table S3. The number of annotated genes in the different species, and the number of STRs in them.

Species	Number of genes	Number of STRs in genes (di/tri)	Number of genes with STRs	Fraction of genes with STRs
Amazon molly	23,627	3,416 (134/2,115)	2,818	0.12
Atlantic cod	23,243	16,848 (5,222/7,535)	7,417	0.32
Haddock	27,437	19,139 (8,206/6,320)	8,200	0.30
Cavefish	23,043	5,591 (1,370/2,338)	4,012	0.17
Fugu	18,525	4,619 (904/1,869)	3,228	0.17

Medaka	19,701	1,766 (78/961)	1,566	0.08
Platyfish	20,381	2,753 (160/1,712)	2,238	0.11
Spotted gar	18,348	2,872 (156/1,693)	2,081	0.11
Stickleback	20,789	3,468 (412/2,809)	2,809	0.14
Tetraodon	19,610	2,689 (376/1,153)	2,223	0.11
Tilapia	21,440	3,224 (191/1,965)	2,619	0.12
Zebrafish	26,498	3,917 (1,156/1,828)	2,740	0.10

Table S4. List of GO terms where haddock and cod differ significantly from most of other species, but not from each other. Terms in bold are where haddock and cod differ significantly from all other species.

GO id	GO name
GO:0000166	nucleotide binding
GO:0003676	nucleic acid binding
GO:0003677	DNA binding
GO:0003700	transcription factor activity, sequence-specific DNA binding
GO:0003723	RNA binding
**GO:0003824**	**catalytic activity**
GO:0004386	helicase activity
**GO:0004672**	**protein kinase activity**
**GO:0004674**	**protein serine/threonine kinase activity**
GO:0004872	receptor activity
**GO:0004930**	**G-protein coupled receptor activity**
GO:0005089	Rho guanyl-nucleotide exchange factor activity
GO:0005216	ion channel activity
**GO:0005488**	**binding**
**GO:0005509**	**calcium ion binding**

GO:0005515	protein binding
GO:0005524	ATP binding
**GO:0005525**	**GTP binding**
**GO:0005622**	**intracellular**
GO:0005634	nucleus
GO:0006355	regulation of transcription, DNA-templated
**GO:0006468**	**protein phosphorylation**
**GO:0006810**	**transport**
GO:0006811	ion transport
GO:0006813	potassium ion transport
**GO:0007165**	**signal transduction**
**GO:0007186**	**G-protein coupled receptor signaling pathway**
GO:0007264	small GTPase mediated signal transduction
**GO:0008152**	**metabolic process**
GO:0008270	zinc ion binding
GO:0016020	membrane
**GO:0016021**	**integral component of membrane**
GO:0016311	dephosphorylation
**GO:0016772**	**transferase activity, transferring phosphorus-containing groups**
GO:0016791	phosphatase activity
GO:0035023	regulation of Rho protein signal transduction
**GO:0035556**	**intracellular signal transduction**
GO:0043565	sequence-specific DNA binding
GO:0046872	metal ion binding
**GO:0055085**	**transmembrane transport**

Table S5. Number of NACHT and FISNA domains in linkage groups in cod

Linkage group	# NACHT	# FISNA
LG01	11	3
LG02	3	1
LG03	1	1
LG04	17	18
LG06	2	1
LG07	7	11
LG08	4	5
LG09	2	3
LG10	1	1
LG11	2	3
LG12	8	6
LG13	1	2
LG15	11	12
LG16	1	1
LG17	4	3
LG18	4	5
LG19	4	4
LG20	1	1
LG21	1	1
LG23	8	8
Total	93	90

Table S6. Number of NACHT and FISNA domains in chromosomes in zebrafish

Chromsome	# NACHT	# FISNA
1	31	31
2	7	3
3	10	7

4	266	261
5	1	1
6	1	1
7	2	0
8	5	4
11	2	1
13	1	2
15	19	18
16	2	1
17	10	10
18	1	0
19	3	3
21	1	1
22	14	14
23	3	3
24	1	0
Total	380	361

Table S7. Number of NACHT and FISNA domains in linkage groups in stickleback

Linkage group	# NACHT	# FISNA
groupI	2	3
groupII	2	1
groupIV	3	3
groupV	2	1
groupVI	5	5
groupVII	2	1
groupVIII	1	0

groupIX	2	2
groupXI	1	1
groupXII	5	3
groupXIII	12	12
groupXIV	5	3
groupXV	1	1
groupXVIII	3	3
groupXIX	2	2
groupXX	1	0
groupXXI	3	5
Total	52	46

Table S8. Number of NACHT and FISNA domains in linkage groups in spotted gar

Linkage group	# NACHT	# FISNA
LG1	1	0
LG2	1	0
LG3	1	1
LG4	3	1
LG5	1	0
LG8	1	0
LG11	1	0
LG13	2	0
LG16	1	0
LG17	1	0
LG19	1	0
LG22	1	0
LG23	1	0
LG24	1	0

LG28	2	0
Total	19	2

Table S9. Number of NACHT and FISNA domains in linkage groups in medaka

Linkage group	# NACHT	# FISNA
1	2	1
2	13	33
3	1	0
4	1	0
7	1	0
8	1	1
9	1	1
10	1	1
13	2	2
14	1	0
16	1	1
18	10	9
19	2	1
20	1	1
22	1	1
24	0	1
Total	39	53

Table S10. Number of NACHT and FISNA domains in linkage groups in tetraodon

Linkage group	# NACHT	# FISNA
2	3	4
3	1	1

4	1	0
5	1	1
7	1	1
15	1	1
17	1	1
18	1	1
Total	10	10
