# Supplementary figures and images for: Genomic architecture of haddock (Melanogrammus aeglefinus) shows expansions of innate immune genes and short tandem repeats
Source: BMC Genomics. 2018 Apr 10;19:240. doi: 10.1186/s12864-018-4616-y (PMC5894186; doi:10.1186/s12864-018-4616-y)

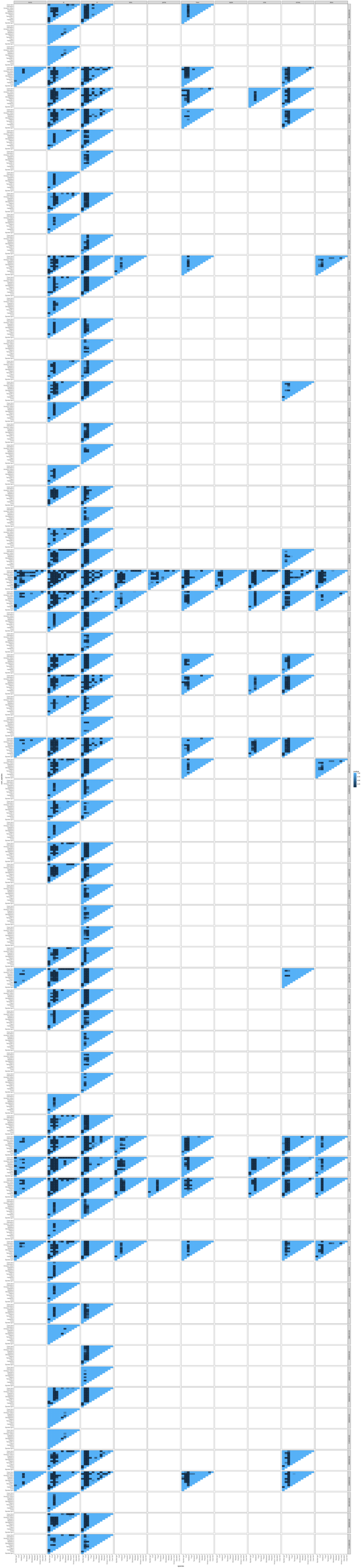

Supplement: Supplementary file 2 — Figure S1. Pairwise Fisher’s exact test between gene ontology (GO) groups and species. Significant differences were found in 74 of 2748 GO groups, i.e. one or more species had significantly higher proportion of genes with STRs in a GO group that other species as found by Fisher’s exact test. In white and light blue areas there are no significant differences, but in dark blue areas there are significant differences between two species. (PDF 244 kb) [file 12864_2018_4616_MOESM2_ESM.pdf]

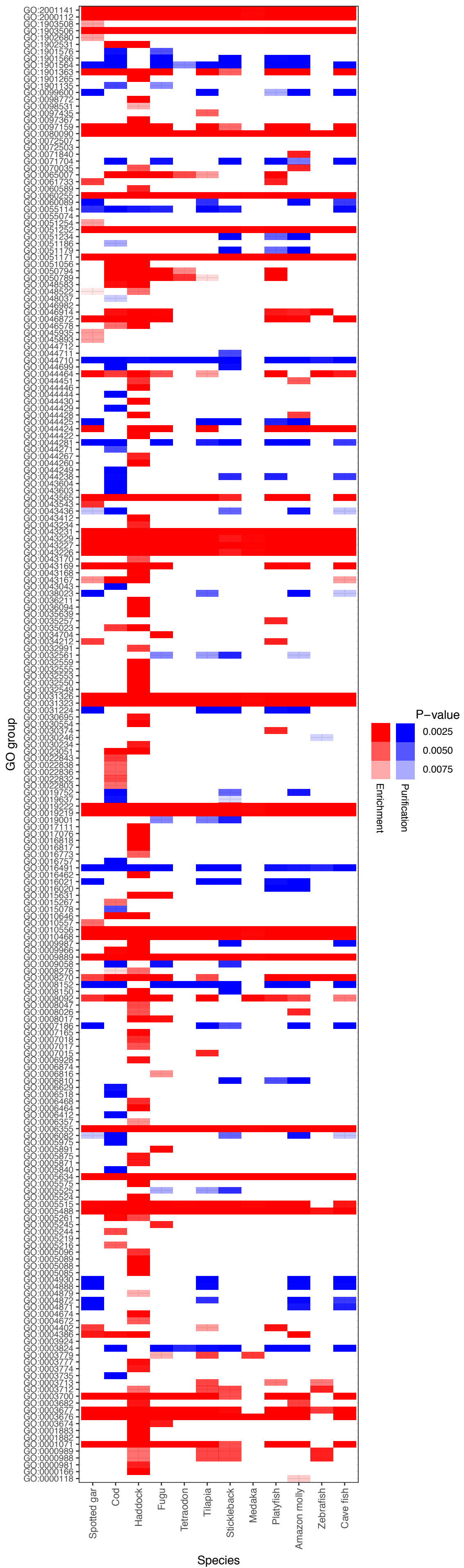

Supplement: Supplementary file 3 — Figure S2. The terms that are significantly enriched for genes with trinucleotide tandem repeats in different species, those repeats that can vary in length without causing frameshifts in the protein. Only tests with P < 0.01 are colored. Red signifies enrichment, more trinucleotide repeats than expected, and blue purification, less than expected. The P-value is signified with color intensity with more bland color being less significant. White areas have no significant differences. (PDF 39 kb) [file 12864_2018_4616_MOESM3_ESM.pdf]
